# Supplementary material for: Determining the Effectiveness of Saccharomyces cerevisiae as a Postbiotic in Mass-Reared Acheta domesticus (House Cricket)
Source: Insects. 2025 Jul 9;16(7):702. doi: 10.3390/insects16070702 (PMC12296080; doi:10.3390/insects16070702)
Supplement: Supplementary file 1 [file insects-16-00702-s001.zip › insects-3654735-supplementary.pdf]

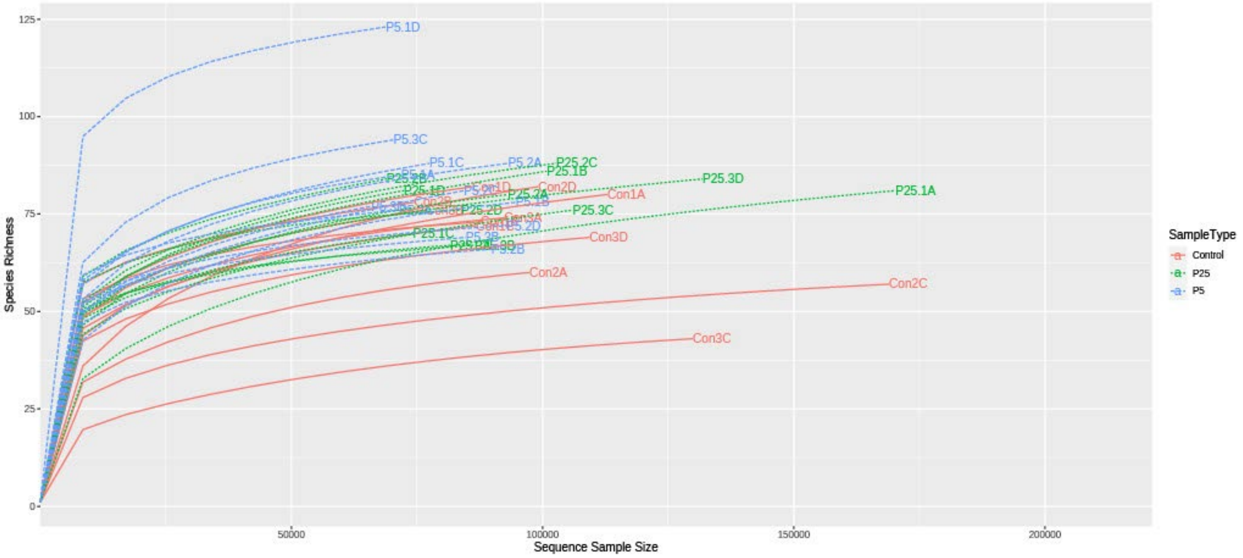

Figure S1: Rarefaction curve for 16S data

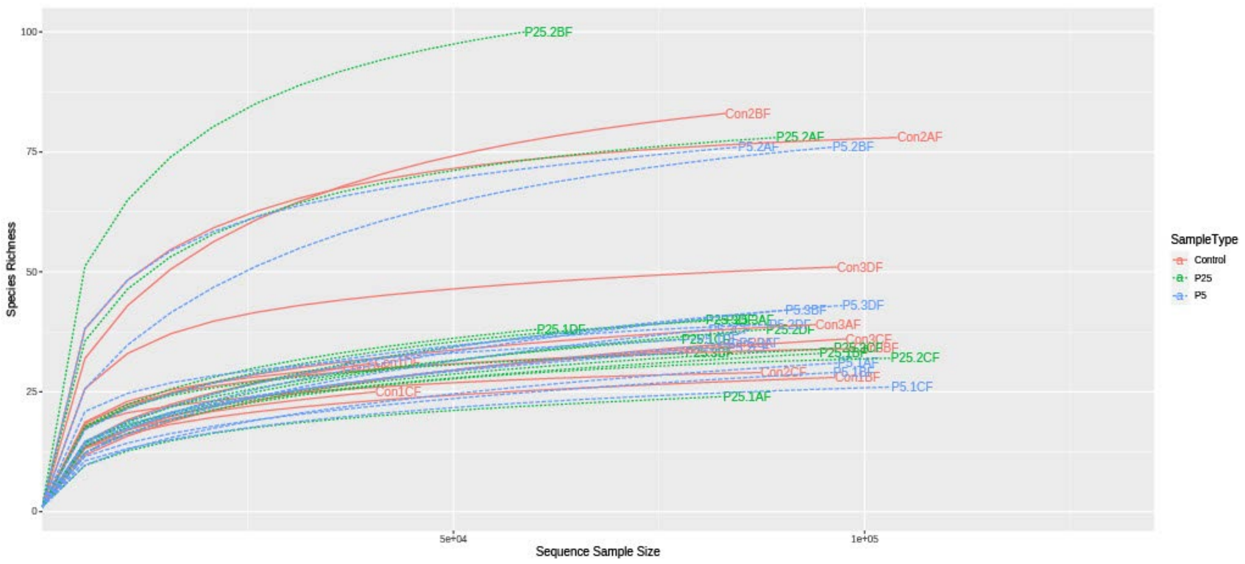

Figure S2: Rarefaction curve for 18S data

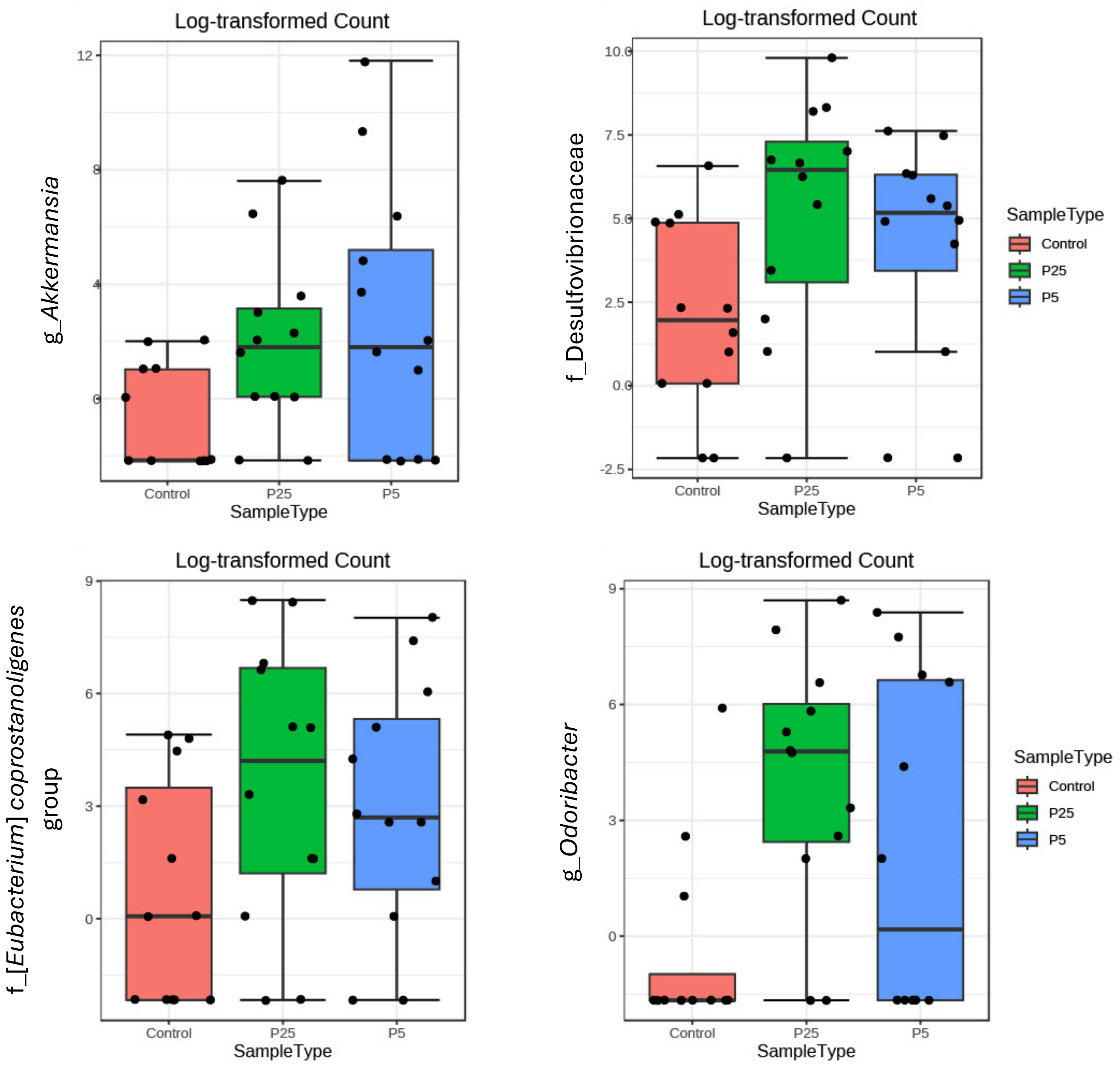

Figure S3: Select single-factor analysis plots for 16S amplicon data. Box and whisker plots show significant differences between the three diet groups for select OTUs. A) *Akkermansia* genus (p=0.004, FDR=0.03). B) *Desulfovibrionaceae* family (p<0.001, FDR=0.01). C) [*Eubacterium*] *coprostanoligenes* group family (p=0.002, FDR=0.02). D) *Odoribacter* genus (p<0.001, FDR=0.02).

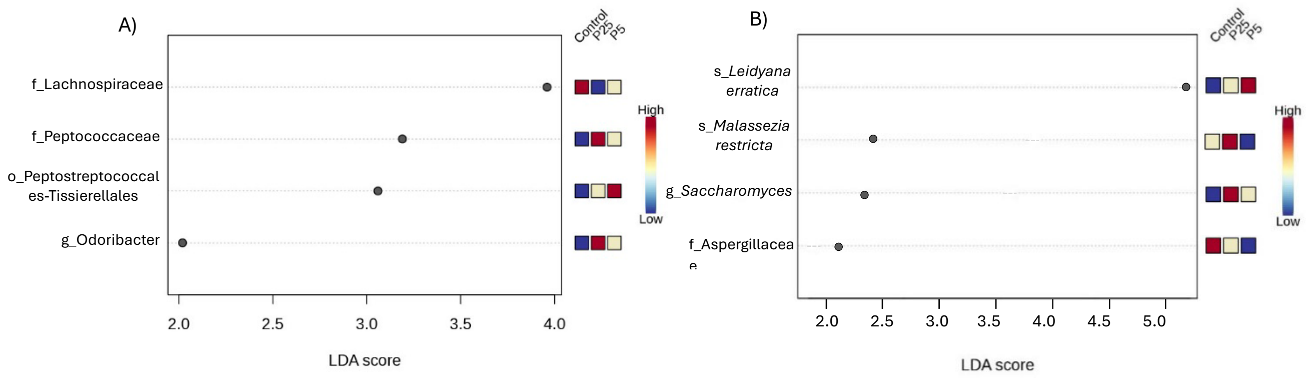

Figure S4: LEfSe LDA plots for a) 16S and b) 18S data. Plots display significant features when analyzed using a p value <0.05 and an LDA score of >2.0.

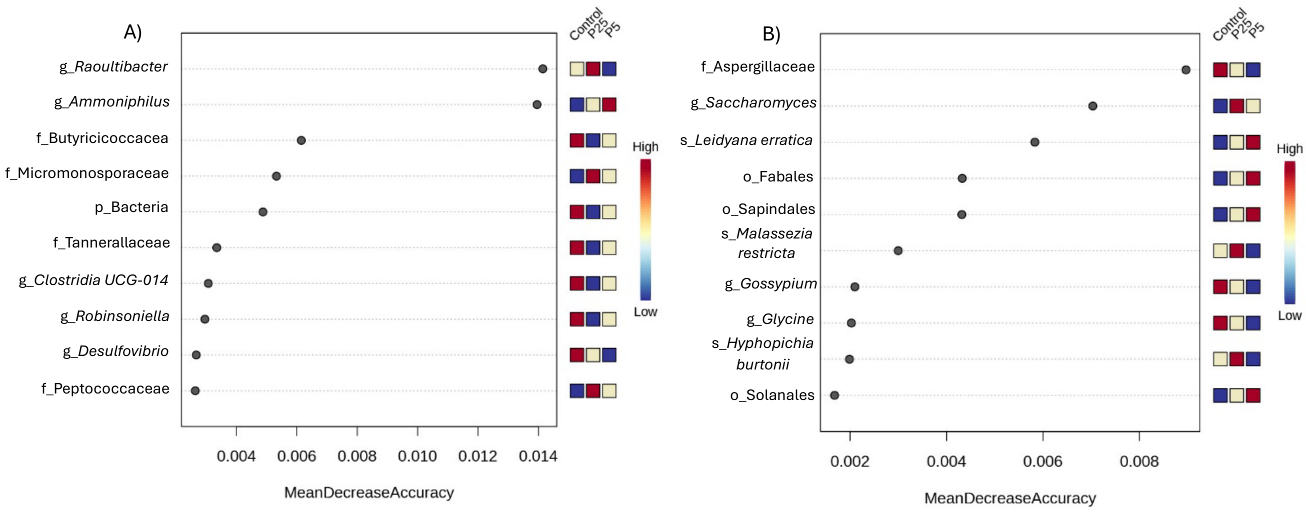

Figure S5: Random forest plots of the top 10 most important features for the a) 16S and b) 18S data.

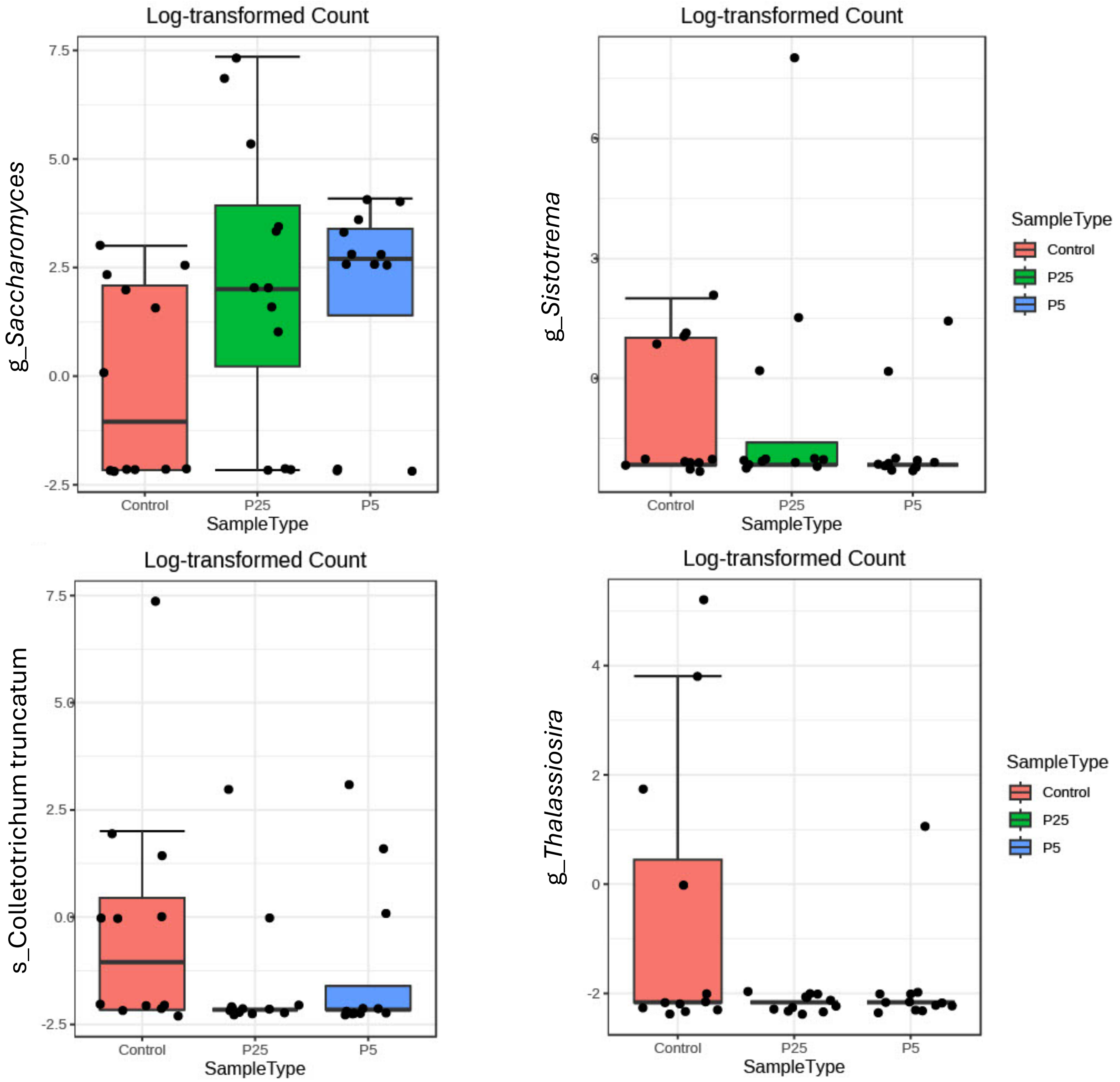

Figure S6: Select single-factor analysis plots for 18S amplicon data. Box and whisker plots show significant differences between the three diet groups for select OTUs. A) *Saccharomyces* genus ($p<0.001$, FDR=0.003). B) *Sistotrema* genus ($p=0.002$, FDR=0.03). C) *Colletotrichium truncatum* ($p=0.002$, FDR=0.03). D) *Thalassiosira* genus ($p<0.001$, FDR=0.01).
